# Interaction of Surface-Modified Alumina Nanoparticles and Surfactants at an Oil/Water Interface: A Neutron Reflectometry, Scattering, and Enhanced Oil Recovery Study

**DOI:** 10.1021/acsami.2c02228

**Published:** 2022-04-20

**Authors:** Wafaa Al-Shatty, Mario Campana, Shirin Alexander, Andrew R. Barron

**Affiliations:** † Energy Safety Research Institute (ESRI), Swansea University, Bay Campus, Swansea SA1 8EN, U.K.; ‡ Laboratory and Quality Control Department, Basrah Oil Company, Bab Al Zubair, Basrah 21240, Iraq; § Science and Technology Facilities Council (STFC), ISIS Neutron and Muon Source, Rutherford Appleton Laboratory, Didcot OX11 0QX, U.K.; ∥ Arizona Institute for Resilient Environments and Societies (AIRES), University of Arizona, Tucson, Arizona 85721, United States; ⊥ Department of Chemistry and Department of Materials Science and Nanoengineering, Rice University, Houston, Texas 77005, United States; # Faculty of Engineering, Universiti Teknologi Brunei, Bandar Seri Begawani BE1410 Brunei Darussalam

**Keywords:** oil, water, interface, neutron reflectometry, functionalization, nanoparticles

## Abstract

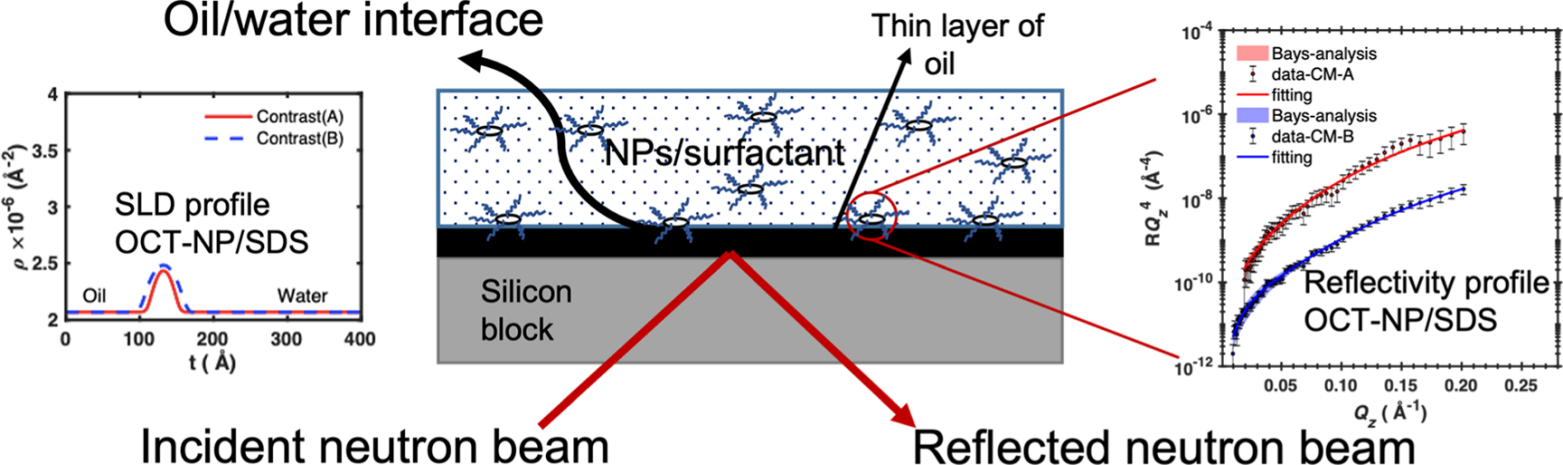

The evaluation of the mechanism of nanoparticle (NP)/surfactant complex adsorption at the critical oil/water interface was studied. A sophisticated technique (neutron reflectometry) was used to give a unique insight on NP/oil interactions in oil recovery systems. Herein, the adsorption of two modified alumina NPs with different degrees of hydrophobicity [hydrophilic = 2-[2-(2-methoxyethoxy)ethoxy]acetic acid and hydrophobic = octanoic acid (OCT)] stabilized with two different surfactants were studied at the oil/water interface. A thin layer of deuterated (D) and hydrogenated (H) hexadecane (contrast matching silicon substrate) oil was formed on a silicon block by a spin coating freeze process. The distribution of the NPs across the oil/water interface with the CTAB surfactant is similar between the two systems. NPs coated with CTAB have more affinity toward the oil/water interface, which explains the oil recovery increase by around 5% when flooding the core with the OCT-NP/CTAB system compared to the surfactant flooding alone. These results suggest that the NP/surfactant complexes can have potential usage in EOR recovery applications.

## Introduction

The observation that nanoparticles (NPs) readily adsorb at interfaces has led to their potential large-scale application in enhanced oil recovery (EOR).^1−3^ A key to this use is an understanding of the relationship between a particular NP’s displacement properties and its structure and surface functionality.^4−6^ Despite the extensive literature dealing with NP adsorption at air/liquid, liquid/solid, and liquid/liquid interfaces, attention has only recently moved to studying the conformation of NPs upon adsorption.^7,8^ This has been due to the lack of suitable techniques to probe such buried interfaces.^9,10^ Unlike other experimental techniques, neutron reflectometry (NR) provides structural information on interfaces (air/liquid, liquid/solid, and liquid/liquid).^11^ Eunhyea et al. established that neutron reflectivity experiments of the interfacial interaction of aqueous solutions of silica NPs with flat silica surface allowed for the determination of parameters such as the thickness of NP layers and NP particle size aggregation (size of the aggregates and particle distribution at the interface).^11^ The reflectometry technique comprises an incoming neutron beam (from a neutron source) impinging a flat surface, from which the reflection is measured in terms of scattering length density (SLD) as a function of depth in the sample.^12^ The reflectivity profile, thus acquired, is used to establish the materials’ composition, thickness, periodicity, and the roughness of the thin film, which is layered on the substrate being examined.^13^

Techniques that have been used to determine the particle size aggregation in colloidal systems such as NPs in solution are dynamic light scattering (DLS) and quartz crystal microbalance (QCM). DLS measurements provide information on the particle size,^14,15^ while QCM provides information on colloidal particles that are removed or deposited onto a surface.^16,17^ Researchers have used both techniques as a complementary method to investigate the aggregation and deposition kinetics of a colloidal system in an aqueous solution.^18,19^ In comparison to these methods, neutron reflectivity is able to provide supplementary information on particles’ clusters in solution and materials deposited onto a surface,^20,21^ most importantly the structure of the materials under study as they are deposited onto a surface.^12,22^ Atomic force microscopy (AFM) is commonly employed to determine the particle/surface interaction but only under ideal conditions; by contrast, NR is more comparable to the performance of a real-world system, making it ideal as a method for studying multi-phase systems with buried interfaces.^21^

A range of colloidal systems have been investigated by NR (including surfactants, polymers, and biomolecules^20,23−26^) in order to investigate the structures of proteins or amphiphilic particles’ monolayers on solid substrates or at an air/water interface as well as the interdiffusion in a thin polymeric film.^27,28^ To date, however, NR has not been used to study the interaction of the NPs with a surfactant for oil recovery applications. This study is the first to address the aggregation, destabilization, and stabilization of NP/surfactant combinations at an oil/water interface.

Herein, we use NR to elucidate the behavior of the different surface-modified alumina NPs dispersed in surfactant solutions. These alumina surfaces have been modified with carboxylic acids, 2-[2-(2-methoxyethoxy)ethoxy]acetic acid (MEEA), and octanoic acid (OCT) to exhibit different degrees of hydrophobicity.^29^ The rational for choosing two different functionalities with different hydrophobicities is to determine whether the degree of wettability affects the NP adsorption on the oil interface and cause the removal of oil from the substrate (i.e., reservoir mineral surface). In addition to the two functionalities of the NPs, both cationic and anionic surfactants were used as a stabilizer, that is, hexadecyltrimethylammonium bromide (CTAB) and sodium dodecyl sulfate (SDS), respectively. Their choice was due to their current ubiquitous use in oil recovery.^30^ The focus of the study is to determine the interaction and the distribution of the modified alumina NPs at the oil/water interface, as well as their interaction with surfactants. The adsorption and volume fraction of the NP/surfactant complexes at the oil (hexadecane) interface were calculated using the SLD.

## Experimental Section

### Materials

Aluminum oxide NPs (13 nm, Aeroxide-Alu), MEEA, OCT, CTAB, sodium dodecyl sulfate (SDS), 2-propanol, *n*-hexadecane, *d*_34_-hexadecane, toluene, and ethanol were purchased from Sigma-Aldrich and used as received except *n*-hexadecane, which was purified by passing it through an alumina column (three times) before its use to remove all impurities. Distilled water (18 MΩ·cm; Millipore) was used throughout the experimental process. D_2_O was obtained from Cambridge Isotopes Laboratories (>98 atom % D). Deuterated CTAB and deuterated SDS were obtained from Santa Cruz Biotechnology and used as received. For oil displacement experiment, two sandstone rock samples from Basra reservoir well were used, and selected properties are provided in Table 1. The viscosity and density of brine and oil were measured at 10, 20, and 30 °C (Table S1, see the Supporting Information). The covalently functionalized carboxylate NPs, MEEA-NPs, and OCT-NPs were synthesized using previously reported procedures.^29,31,32^

**Table 1:** Reservoir Rock Properties

sample	depth (m)	length (cm)	area (cm^2^)	air permeability, *K*_a_ (md)	porosity, φ (%)	pore space, *V*_p_ (cm^3^)
R1	2806.1	5.48	11.46	968	27	17.79
R2	2807.13	5.5	11.34	1991	27.4	17.09

### Fitting for NP–Surfactant Mixtures at the Oil/Water Interface

The neutron reflectivity experiment at the oil/water interface was performed using the methodology developed by Zarbakhsh et al.^33^ using the INTER reflectometer at ISIS, Ral, Didcot, UK.^34,35^ The oil layer was spin-coated onto a hydrophobic silicon block modified by a layer of trimethylchlorosilane silane as previously detailed.^36^ The thickness of the oil film is ∼2.1 μm based on the method that is fully descripted in detailed.^36^ The oil layer was then sandwiched between the silicon layer and the aqueous phase. The samples were allowed to equilibrate for at least 45 min prior to measurement. Measurements were performed at two incident angles, 0.7 and 1.4°, and stitched together after subtracting the wavelength-dependent oil transmission. The data was analyzed using Rascal and then replotted by MATLB. The interface is divided into discrete layers, each characterized by roughness (σ), thickness (*t*), and SLD (ρ), which is a function of layer composition as shown in eq 1, where Φ_*i*_ is the volume fraction of species *i*.
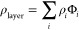
1

The reflectivity is then calculated using the optical matrix method^37^ and compared to the experimental data. The routine is iterated until reaching a least-squared minimization. The adsorbed amount (Γ), expressed in mg·m^–2^, can be calculated using eq 2, where *t* is the fitted layer thickness (determined from the fitting routine) and *d* is the density expressed in g·m^–3^.
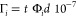
2

The SLD values for all components used in the study are shown in Table S2 (see the Supporting Information). The two NPs (MEEA-NPs and OCT-NPs) were studied independently in the presence of both CTAB and SDS surfactants at critical micelle concentrations (CMCs). In all cases, both the oil and the aqueous phase were contrast-matched to silicon (see the Supporting Information for calculations), and each system was characterized at two different surfactant contrasts: (A) a mixture of deuterated and hydrogenated (non-deuterated) surfactant to match the SLD of silicon and (B) just the deuterated surfactant. The contrast schemes are depicted in Figure S1 (see the Supporting Information). Knowing the NP–surfactant solution density from pycnometer measurements (Table S1, see the Supporting Information) and the SLD of the NPs from small-angle neutron scattering (SANS) measurements (4.8 × 10^–6^ Å^–2^),^29^ contrast (A) enables us to calculate the adsorbed amounts of NPs at the interface. In comparison, contrast (B) shows an increase in scattering intensity due to the presence of deuterated surfactant: the more the increase in signal, the higher the amount of surfactant at the interface. In both cases of OCT-NP, more increase in scattering was observed with contrast (B), indicating a larger amount of adsorbed surfactant compared to MEEA-NPs. In all cases, both contrasts were co-fitted to a single model.

### NP and Surfactant Characterization

SANS measurements were carried out on Larmor at ISIS, Didcot,^38^ UK. Larmor is a fixed-sample detector instrument that uses neutrons with wavelength 8 Å and two samples of the placement detector (1.2 and 8 m) to provide a *Q* range of 0.002–0.4 Å. All samples were measured in 2 mm path length rectangular quartz cells in D_2_O. The raw SANS data were normalized by subtracting the scattering of the empty 2 mm cell and D_2_O (SLD, p = 6.33 × 10^–6^ Å^–2^) as a solvent background at 25 °C. Each sample solution was prepared by first making the surfactant solution at CMC 0.9 mM (0.32 g L^–1^) and 2.8 mM (2.36 g L^–1^) for CTAB and SDS, respectively, using 10 mL of D_2_O (taking into account the density of heavy water) and stirring for 24 h to reach equilibrium. Then, each of the NPs (0.05 g, 0.5 wt %) was added into surfactant solutions and left to stir for another 24 h to create homogeneous surfactant/NP dispersions. The dispersions were transferred into 2 mm rectangular quartz cell cuvettes with lid and placed into a SANS chamber where measurements were carried out. Data reduction used the Mantid data analysis package^39^ program, and fitting of SANS was carried out using the SASVIEW program.^40^ Zeta potentials (ZPT) were used to determine the charge of the tested system in dispersed solutions. For charge measurements, (0.5 wt.%) the concentration of each NP sample was dissolved in 10 mL of deionized (DI) water or isopropanol (considering the density, in the case of hydrophobic OCT-NPs) and left magnetically stirred for 24 h to create homogeneous dispersion. For the charge of surfactants (at CMC) of each surfactant CTAB and SDS, the samples were dissolved in 20 mL of DI water and left for 24 h to reach equilibrium. The samples of the NPs (0.5 wt %) were then weighed and added onto 10 mL of the surfactant solutions and left for another 24 h to create a homogeneous dispersion. The analysis was performed using a Zetasizer Nano Zs equipped with a He–Ne laser operating at a wavelength of 633 nm at 20 °C with a 120 s equilibration time and 173° scattering angles. Data processing was performed by the Zetasizer software. The data was the average of five measurements. Surface tension (SFT) and interfacial tension (IFT) on hexadecane oil have been measured by a collection of time-dependent methods using a drop shape analyzer (Krüss) at an ambient condition. A disposable plastic syringe was filled with the NP/surfactant solution, placed in a chamber, and loaded gently. All the SFT and IFT values were an average of three repeated measurements. The IFT values between the NP/surfactant solution and hexadecane oil were measured using the same method with the filled syringe immersed into the hexadecane oil phase. For both SFT and IFT measurements, the syringe was calibrated before each test and then analyzed with ADVANCE software. The IFT value was obtained by fitting the Young–Laplace equation to the contour profile of 4.0 μL droplets.

### Fluid’s Formulation, Reservoir Rock Cleaning, and Modification

NP–surfactant mixtures (MEEA-NPs and OCT-NPs with CTAB and SDS) were formulated by preparing a surfactant solution at CMCs 0.9 mM (0.32 g L^–1^) and 2.8 mM (2.36 g L^–1^) for CTAB and SDS, respectively, by stirring for 24 h to reach equilibrium. Following that, the NP powder (0.5 wt %) was added to each of the surfactant solutions and left to stir for 24 h at 25 °C to create a homogeneous dispersion (Figure S2, see the Supporting Information). Brine solution was made at 20 wt % NaCl in DI water. The reservoir rocks were cleaned via Soxhlet extraction using toluene for 2 weeks to remove all organic compounds. The rock samples then dried at 60 °C in air. The samples were further cleaned by DI water to remove salt twice daily and at each time tested for the presence of salts with AgNO_3_. After removing all ions/salts from the reservoir rock, it is then dried with an air oven at 60 °C for 7 h.

### Core Flooding Experiment

Figure S3 (see the Supporting Information) shows a schematic representation of the core flooding experimental setup.^29^ The aim of the core flooding tests was to evaluate the capability of the functionalized alumina NPs as potential agents of enhancing oil recovery in reservoir rocks after flooding with high-salinity brine solution. At the beginning of each test, a core was fully saturated with brine solution (20 wt % NaCl) in a close high-pressure stainless steel cylinder at 1500 psi for 2 days. The core flooding system is characterized with an oven for adjusting the temperature and three piston cylinders for accommodating the injection fluids. Additionally, a core holder was used to enable the system to perform different injection schemes. The first accumulator cylinder was filled with brine, while the second and third cylinders were used for oil and nanofluid testing, respectively. Each core sample was cleaned after every use by Soxhlet extraction (vide supra). A series of core-flooding experiments were performed to evaluate the effect of the functionalized alumina (MEEA-NPs and OCT-NPs) with a surface-active agent (either CTAB or SDS) present on flooding performance. The system was pressurized to 1500 psi, supported with 100 psi as a backpressure. The procedure for oil flooding test includes air evacuation, initial saturation of the core with brine (20 wt % NaCl), and then oil flooding until the water saturation was reached.^41,42^ An air compressor pump was used for pumping the injection fluids from accumulator through the core flooding system. Medium oil was injected at a flow rate of 0.3 mL/min. The system was then aged for 2 weeks to establish equilibrium and attain uniformity. Subsequently, brine injection was continued until the oil amount was 0.05 mL. Afterward, the test/synthesized nanofluid was injected at a flow of 0.3 mL·min^–1^ to recover the remaining oil.

## Results and Discussion

### Reflectivity Profiles

The reflectivity profiles measured for the NP/SDS systems were initially fitted to a single-layer model. The fitting parameters for this were the layer thickness, the volume fractions of NPs and SDS, and 2 (*n* layers +1) interlayer roughness. The reflectivity profiles and best fits are shown in Figure S4a (see the Supporting Information) for MEEA-NP/SDS and Figure S4b for OCT-NP/SDS. The single-layer model provides an adequate fit, but the layer thickness is smaller than expected for the NPs. Thus, the fitted thickness was 48.5 and 38.5 Å for MEEA-NPs and OCT-NPs, respectively. This is different from the expected particle size of around 42 and 400 Å for polar and equatorial radius, respectively, for both NP systems.^29^ This discrepancy should not come as a surprise as we are attempting to represent a layer of ellipsoidal objects as a uniform slab, which is not the ideal case. In the event of a well-organized layer, there should be a gradient of material where the densest part is at the center.

In order to represent the interface with a more realistic model, we have used SANS data (an elliptical NP with a core of 42 × 400 Å)^29^ covered with a homogeneous layer of the surfactant with a thickness of 20 Å (i.e., the shell) corresponding to a fully extended surfactant molecule. Since the shell may not be fully composed of the surfactant, we have introduced a parameter called surfactant coverage to simulate the amount of surfactant on the NP surface (0 = no surfactant and 1 = full coverage). The NP/surfactant complex was then sliced into 10 slices of equal thickness, whereby the thickness *d* was a fitting parameter. The volume fraction of both the core and shell is thus geometrically constrained. The coverage of the NP–surfactant complexes was then fitted by introducing a new fitting parameter: the packing of particles at the interface. Assuming a well-organized layer of circles in a plane, the maximum packing achievable is ∼0.906 (or slightly higher because of the elliptical nature of the NPs and possibly staggering of the NPs). With this geometric model, the fitting parameters included the thickness of each slice, the surfactant coverage in crown, and the sphere packing. In order to simulate a smooth transition between each layer, the interlayer roughness was fixed as half of the fitted layer thickness.

**Figure 1:**
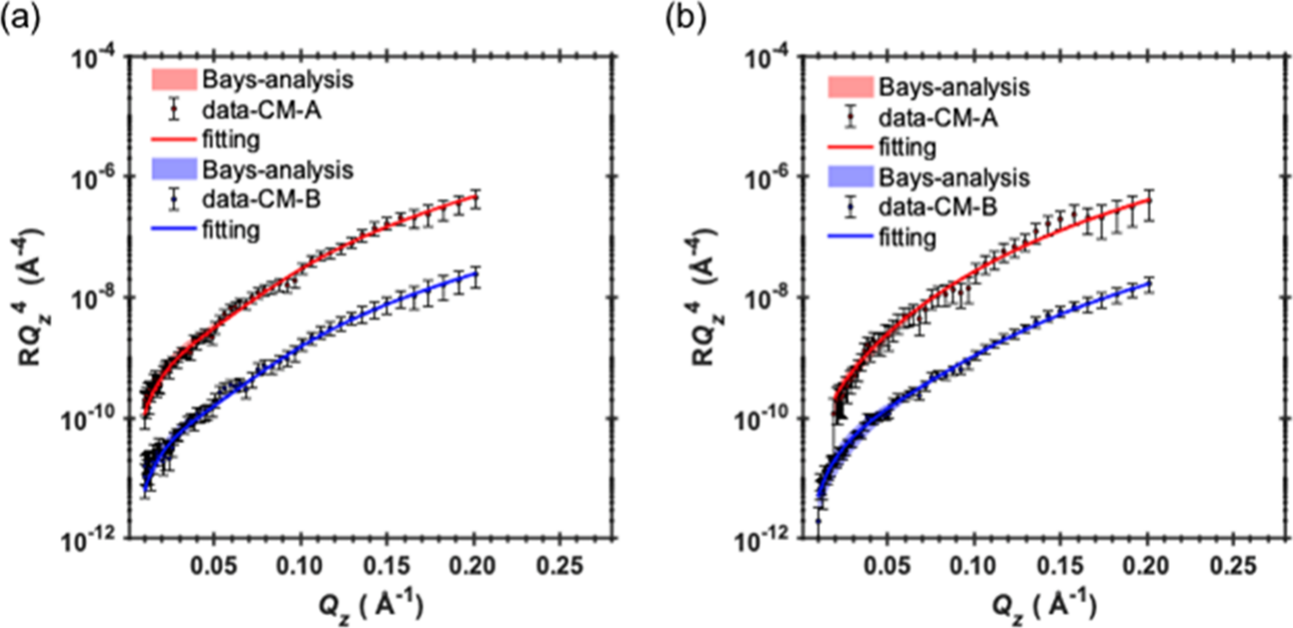
Data and best line fits for the (a) MEEA-NP/SDS and (b) OCT-NP/SDS systems using the disk-like geometric model. The shaded areas correspond to the 95% confidence interval as determined by Bayesian analysis. Contrast (A) is shown in red and contrast (B) in blue. Profiles are offset to visualize the quality of the fit.

Applying this model to MEEA-NP and OCT-NP complexes with SDS, the quality of the fit was not affected. The best fits are shown in Figure 1 for MEEA-NP/SDS and OCT-NP/SDS, respectively. The overall thickness increases, respectively, to 112.8 ± 17 and 63.2 ± 11 Å in both cases by a factor of 2.3–1.6. The thicker interface observed for the MEEA-NP/SDS complexes could be an indication of slight staggering; however, this would not be due to the increased surface packing (within the error for the two systems). Alternative explanations for the observed staggering could be due to the disk-like shape of these particles, the surfactant distribution across the interface appears to be somewhat bimodal (Figure 1), where the top and bottom parts of the interface are surfactant rich and most of the NP resides in the middle part. The adsorption of the near-spherical particles coated with an analogous surfactant shell has been simulated, where the surfactant distribution is more similar to that of the NP (Figures S5–S7, see the Supporting Information). This may well be the case in the present situation as the role of the surfactant if a dispersant; therefore, the formation of smaller complexes is to be expected in the presence of the surfactant. It must be stressed that in both NP–SDS systems, the adsorbed amount of the two components is hardly affected by the shape of the particles; hence, the quantitative interpretation is independent on the choice of the model used (Table S4, see the Supporting Information). In addition, this could very well be for an additional reason: the OCT-NP is hydrophobic, it is more likely in order to force these NPs to disperse in an aqueous solution, and the surfactant covers small particles before agglomerations happened.

The adsorbed amount for both NPs and SDS is shown in Table 2, and detailed layer-by-layer calculations are shown in Tables S3 and S5 (see the Supporting Information). Of the two NPs, MEEA-NP shows a higher adsorbed amount at the interface compared to OCT-NP: 0.76 and 0.42 mg·m^–2^, respectively. What is more interesting is the trend of the surfactant adsorbed in terms of amount. For example, MEEA-NP requires a little surfactant adsorption to become surface active; on the other hand, the OCT-NP/SDS mixture adsorbed at the interface presents a surfactant-rich crown, showing that much more surfactant is required to make the OCT-NP surface active. We have investigated the possibility of free surfactant co-adsorption at the interface. This can be easily modeled by introducing additional fitting parameters, but the increase in quality fit does not justify the use of an increasingly complex model. At this stage, we may not be able to exclude the presence of free surfactant at the oil/water interface, and this experiment does not show any clear evidence of its presence.

**Table 2:** Adsorbed Amount for NPs and Surfactants in the Four Systems Studied^a^

	adsorbed amount, Γ (mg·m^–2^)	
layer	NP	surfactant
MEEA-NP/SDS	0.755 (0.676, 0.852)	0.035 (0.0026, 0.088)
OCT-NP/SDS	0.416 (0.360, 0.495)	0.154 (0.097, 0.247)
MEEA-NP/CTAB	5.850 (5.251, 6.375)	0.384 (0.110, 0.728)
OCT-NP/CTAB	6.207 (5.605, 6.863)	0.674 (0.512, 0.958)

a Surfactant and NPs are shown separately for each system. In all cases, the total optimum adsorbed amount is shown together with the 95% confidence interval in parentheses.

**Figure 2:**
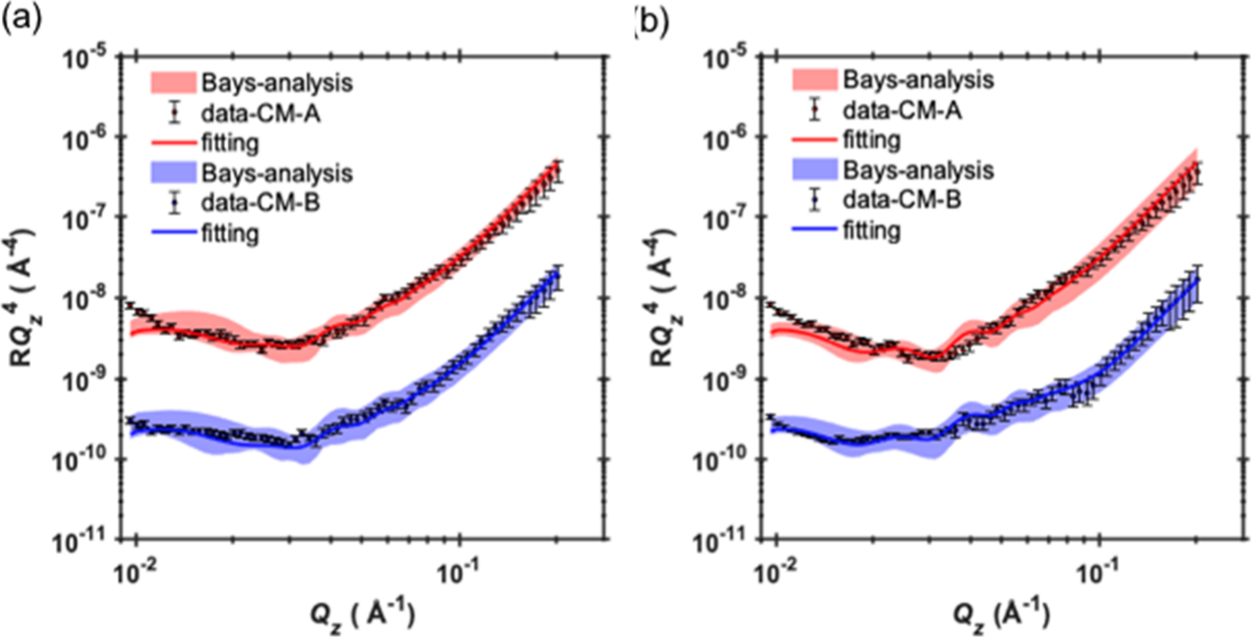
Data and best line fits using three-layer model for (a) MEEA-NP/CTAB system and (b) OCT-NP/CTAB system. Although the fit for contrast (A) is the worst of all reported here, large error bars allow us to describe the main features of the reflectivity curve.

The single-layer model used to describe the NP–SDS systems fails to describe the interface in the presence of NP–CTAB. An example of the poor one-layer fit is shown in Figure S8a (see the Supporting Information). The geometric model also failed to fit the reflectivity profiles; this proves that the adsorption of NPs in the presence of CTAB leads to a more complex interface that cannot be modeled simply in the same way as with SDS. To overcome the bad fitting, a step to gradually increase the complexity was taken as an approach while maintaining to a minimum number of fitting parameters. Initially, a second layer was added to the model to increase its complexity. The fitting parameters included the two-layer thicknesses, the volume fractions of NPs and CTAB in each layer, and three interlayer roughnesses. Again, the best model could not fit the experimental data (Figure S8b, see the Supporting Information); therefore, a further increase in model complexity was required. A three-layer model was found to adequately describe the oil/water interface in the presence of MEEA-NP/CTAB. The fit quality is slightly inferior for OCT-NP/CTAB; an attempt was made to increase the complexity of the model by adding a fourth layer but with little effect on the fitting quality. Another approach taken was to introduce a free surfactant layer in different locations across the interface; however, no noticeable improvement in the quality of the fitting was observed. In summary, the three-layer model presented here is believed to be the best fit for the collected data. The data and best fits for the three-layer model are shown in Figure 2.

In both systems, the interface could be modeled with a monolayer of relatively good coverage (around 45% in both cases), followed by a much thicker secondary layer with around 25% coverage. A thick diffuse layer, with coverage between 3 and 6%, was then observed on the aqueous side of the interface. The roughness between layer 2 and layer 3 is very large (however within less than half of the layer thickness), indicating a gradual transition between the secondary adsorption layer and the diffuse region.

The thickness of the first layer, which represents the most ordered region within the interface, is around 33 ± 9 Å. The thickness of this region is smaller compared to a single NP–CTAB complex; however, the same argument used for the NP–SDS complexes apply here: we are attempting to represent a layer composed of ellipsoidal objects using a single slab. Therefore, one would expect the real thickness of this region to be in line with what reported for the NP–SDS complex.

The thickness of the secondary adsorption layer and the diffuse region (third layer) is significantly thicker than the primary monolayer: for MEEA-NP/CTAB, *t* = 159 ± 11 and 177 ± 16 Å, respectively; similarly, for OCT-NP/CTAB, *t* = 175 ± 6 and 180 ± 13 Å. This shows that the overall thickness of the interface is similar, and it is much broader with CTAB compared to SDS.

The detailed layer-by-layer fitted data are shown in Tables 3 and 4 for both NP/CTAB complexes. The adsorbed amount of NPs in the primary layer for MEEA-NP and OCT-NP is within the error; however, this region contains considerably more CTAB with OCT-NPs (7–13%) compared to MEEA-NPs (2–5%). This is in comparison with what was observed for the NP–SDS complexes, where OCT-NPs at the interface were associated with more surfactant molecules compared to MEEA-NPs (Table S6). The amounts of NPs in the secondary layer are less compared to the primary layer; however, the decrease in the amount of the surfactant between the two regions is much more pronounced, dropping to 0–2%. The same was observed in the diffuse region, where the amount of the surfactant dropped to less than 1.5% in both cases. Overall, the adsorbed amount of NPs at the interface is 5.85 ± 0.58 mg·m^–2^ for MEEA-NPs and 6.21 ± 0.62 mg·m^–2^ for OCT-NPs. The distribution of NPs across the interface is remarkably similar between the two systems, with most of the material (∼67%) contained in the secondary adsorption layer. This is because, despite showing a smaller volume fraction compared to the first layer, it is considerably thicker.

**Table 3:** Layer-by-Layer Detailed Adsorbed Amount Γ for the MEEA–CTAB System^a^

	adsorbed amount Γ (mg·m^–2^)	
layer	MEEA	CTAB
primary layer	1.295 (1.105, 1.478)	0.118 (0.062, 0.170)
secondary layer	3.981 (4.129, 4.477)	0.177 (0.052, 0.314)
diffuse layer	0.574 (0.346, 0.784)	0.090 (0.000, 0.274)

a In all cases, the optimum adsorbed amount is shown together with the 95% confidence interval in parentheses.

**Table 4:** Layer-by-Layer Detailed Adsorbed Amount Γ for the OCT-NP/CTAB System^a^

	adsorbed amount Γ (mg m^–2^)	
layer	OCT-NP	CTAB
primary layer	1.197 (1.027, 1.422)	0.333 (0.224, 0.456)
secondary layer	4.152 (3.957, 4.328)	0.177 (0.141, 0.265)
diffuse layer	0.858 (0.621, 1.114)	0.164 (0.146, 0.237)

a In all cases, the optimum adsorbed amount is shown together with the 95% confidence interval in parentheses.

The amount of the surfactant at the interface differs greatly between the two systems, with OCT-NP–CTAB showing more surfactant at the interface compared to MEEA-NP/CTAB. This can also be visually seen from the increase in scattering between contrast (A) and contrast (B), which is much more pronounced for MEEA-NPs and OCT-NPs. It is noteworthy that not only the amount of surfactant differs between the two systems but also its distribution across the interface. The amount of both NP and CTAB found in the secondary layer is fairly similar between the two systems; therefore, it can be inferred that the structure of these two regions is very similar in the two systems. In the case of OCT-NP/CTAB, there is however a considerable amount of surfactant in the first layer, ca. 3 times higher than the MEEA-NP/CTAB system.

The difference in CTAB adsorbed amount in the primary layer is the most striking difference between the two systems and is in analogy with what reported for the NP–SDS systems. Similarly, to OCT-NP/SDS, the adsorbed amount ratio of NP to surfactant is around 3:1. The amounts of NPs and SDS in solution are 5.00 and 2.50 mg·mL^–1^, respectively, and the ratio 3:1 is not too different from the stoichiometric ratio in solution. However, the amount of CTAB in solution is considerably lower: there is only 0.33 mg·mL^–1^ as opposed to 5.00 mg·mL^–1^ of NPs when preparing the solution, very far from the 3:1 NP to surfactant found in the primary monolayer. There are several possible explanations for this discrepancy:

First, surfactants preferentially adsorb to some NPs. This could be because of size distribution: smaller NPs have a higher surface-to-volume ratio, adsorb more surfactant molecules, and become more surface active; hence, a higher fraction of the surfactant at the interface. This could also be caused by differences in NP coatings, causing some NPs to have a higher affinity to surfactant than others.

Second, surfactants are not effective at dispersing the NPs. Large aggregates may be present in solution, and the surfactant may not be ready to separate the individual NPs effectively. In this case, the free NPs have access to a small fraction of surfactant and become readily surface active, reaching the interface first, forming the primary monolayer on their own resulting in a higher NP-to-surfactant ratio. The larger aggregates are characterized by a slower Brownian motion and would reach the interface after the smaller aggregates; these are surfactant-rich complexes and would therefore form the secondary adsorption layer. This would also explain why the secondary layer forms even when the primary layer is far from full coverage: these larger aggregates would act as a steric barrier for other NPs in solution.

**Figure 3:**
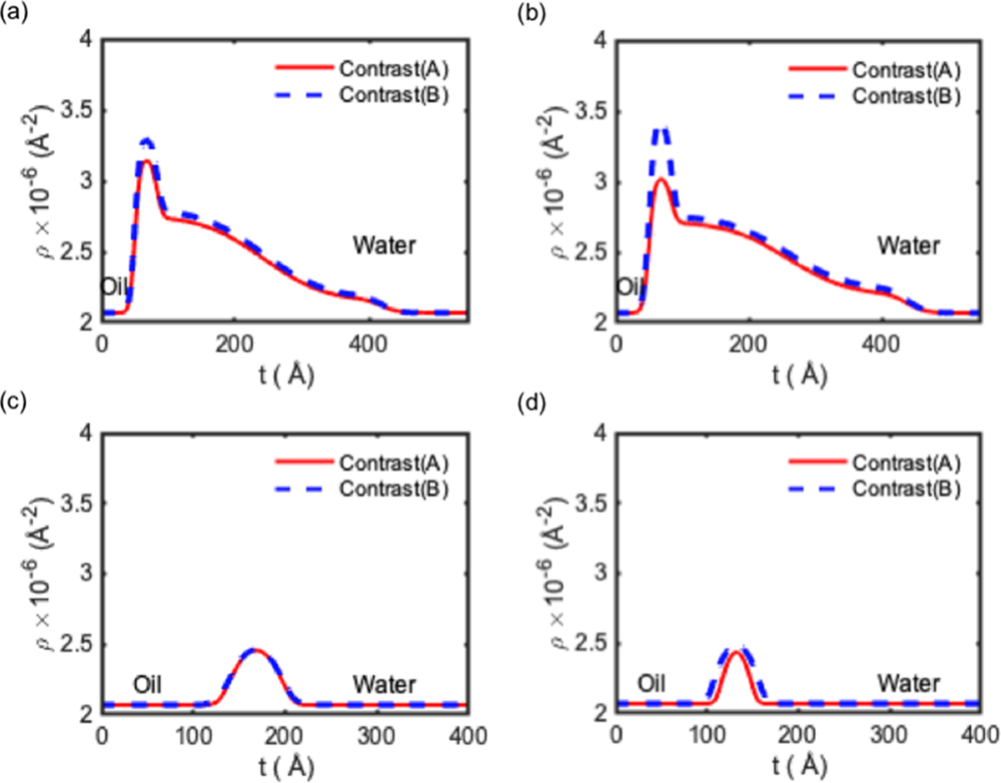
SLD profiles for all samples measured in the experiment. Contrast (A), with the CMSi surfactant, is shown in red, while contrast (B), with *d*-surfactant, is shown in blue. Note that in all cases, contrast (A) has a lower signal compared to contrast (B).

**Figure 4:**
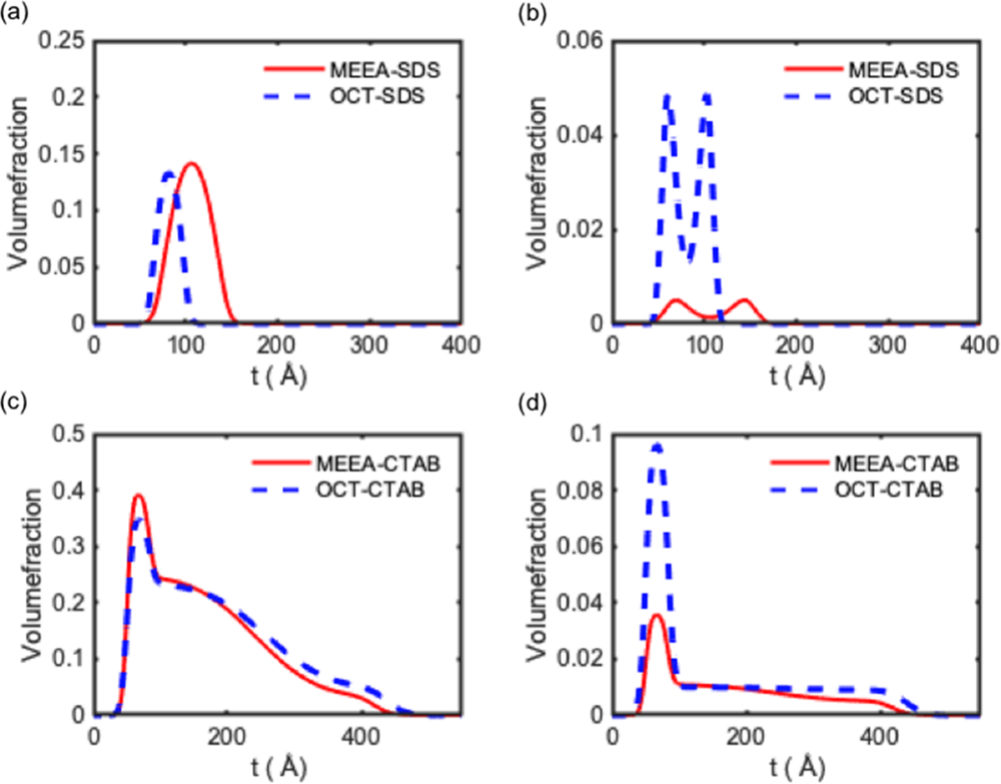
Distribution of NPs (a,c) and surfactant (b,d) across the interface for both systems for NP/SDS systems (a,b) and NP/CTAB systems (c,d). The scaling for the NP and surfactants is different to facilitate visualizing the higher amount of adsorbed NPs compared to the surfactant. a—top left: Φ of NP; b—top right: Φ of SDS; c—bottom left: Φ of NP; and d—bottom right: Φ of CTAB.

Third, the NP–surfactant complexes adsorb at the interface together with the free surfactant. This is proving difficult to verify as the fits are inconclusive on the matter. The SLD and volume fraction profiles are shown in Figures 3 and 4, respectively.

### SANS Measurements

**Figure 5:**
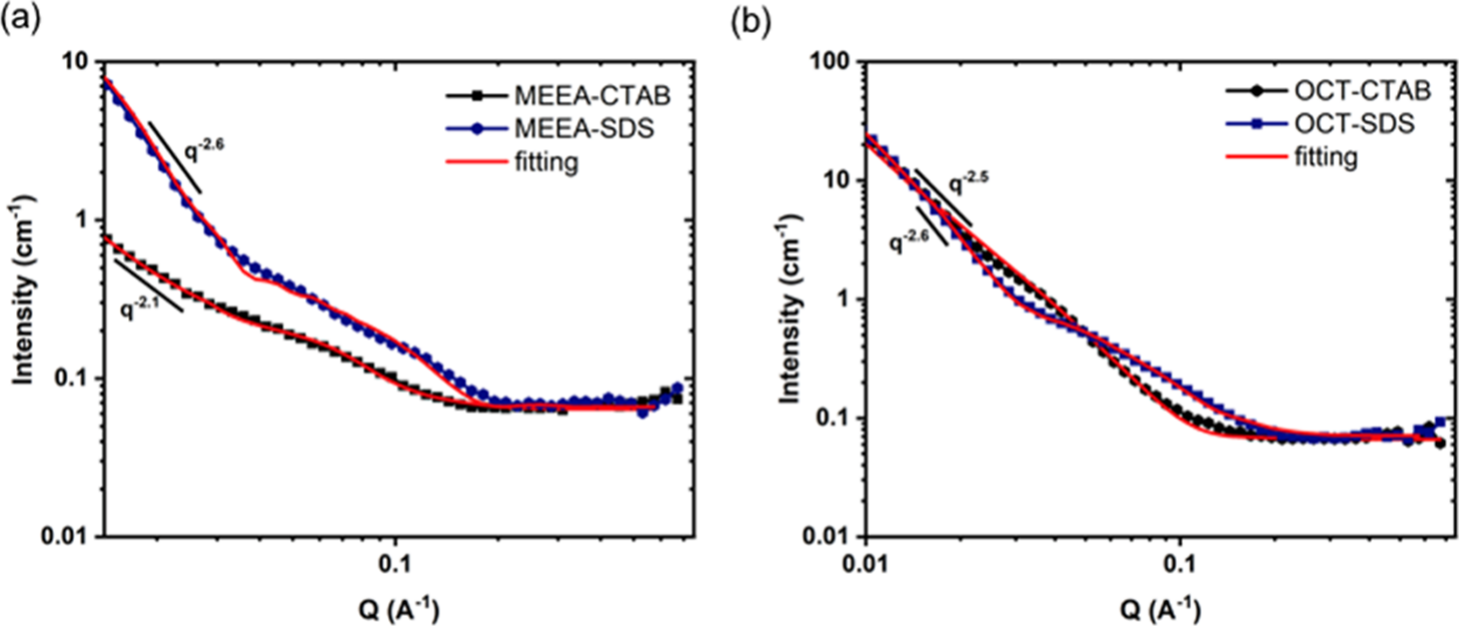
SANS scattering patterns form 0.5 wt/v % of (a) MEEA-NPs and (b) OCT-NPs at a CMC of surfactant solution (CTAB and SDS) at 25 °C in D_2_O.

The SANS data were collected for 0.5 wt % NPs (MEEA-NPs and OCT-NPs), which dispersed in the surfactant solution (CTAB and SDS) at CMC. The SANS scattering patterns are shown in Figure 5. As can be seen from the scattering patterns, the system appears to be bimodal with higher *Q* range corresponds to the scattering of the surfactants and the lower *Q* range to the NPs. Due to the complexity of the scattering data, we fit the data with the power law to understand the system. The power low shows that both NPs with both surfactants have a *Q* slope of around 2.1–2.5 at lower *Q*, which indicates the scattering from a two-dimensional subject such as a plate or an ellipsoidal.^29^ There is one possible explanation for this bimodal system, and this can be due to NPs being coated with both unimeric and surfactant micelles. The data at the high *Q* region shows a similar shape to the surfactant micelles,^43^ which could be due to scattering from the micelles around the NPs. However, the data from the lower *Q* region has a pattern similar to NPs, which were fitted to an ellipsoidal structural (42 and 400 Å for polar and equatorial radius),^29^ which indicates that surfactants have coated NPs as unimers rather than micelles. The estimated size using Gunner–Debye model is ∼22 nm, which are small enough that would not to block the core pores.

### IFT, SFT, and Oil Recovery

The pendent drop method was performed in order to investigate the effect of NPs in combination with surfactants (SDS and CTAB) on the IFT values. Table 5 and Figure S10 (see the Supporting Information) show the static and dynamic measurement between NPs with SDS and CTAB in *n*-hexadecane. It shows that using dispersed NPs in surfactant solutions leads to a significant decrease in IFT. The dynamic measurements were conducted on NPs in surfactant solution over 120 s in *n*-hexadecane and are shown in (Figure S10, see the Supporting Information). As can be seen, the IFT is quite stable with time. Our previous research showed that both NPs are able to reduce the IFT of hexadecane from 51 mN/m up to 40.7 and 45.1 mN/m for OCT-NPs and MEEA-NPs, respectively.^31^ By comparing the IFT reduction of NPs alone and with surfactant addition shown in Table 5, it can be observed that there is significant reduction of IFT of hexadecane oil due to the presence of surfactants, which indicates that the interaction between the surfactant and NPs increases the reduction of IFT.^44^ In order to find the charge of the tested system and confirm the NP/surfactant interactions, the zeta potential has been used. It was found that un/modified alumina NPs are positively charged in the absence of both surfactants. As has been discussed earlier in the study, MEEA-NPs are superhydrophilic, therefore stable in water (ZPT = 40);^29^ however, OCT-NPs are superhydrophobic, therefore are not dispersible in water (ZPT in ethanol = 30).^29^ Upon the addition of both surfactants, OCT-NPs become fully dispersible in water with ZPT ±35 depending on the charge of the surfactants, which indicates a stable dispersed system.^45^ As CTAB is a cationic surfactant with positive charge, the charge of the NPs remains positive and stable for the duration of the experiment; however, it altered to negative after the addition of SDS solution (due to the fact that SDS is an anionic surfactant). These results are in line with the direct visual observation result, which shows dispersed solutions up to 1 day (Figure S2, see the Supporting Information).

**Table 5:** IFT, SFT, and Zeta Potential of NPs with CTAB or SDS Surfactants, the Data All Within ±0.5 Error

materials	IFT (mN/m) at 20 °C	SFT (mN/m) at 20 °C	zeta potential at 20 °C
hexadecane	50.52		
CTAB	5.27	36	+53
SDS	8.8	34	–31
unmodified alumina	41.3		39.6
MEEA-NP/SDS	8.9	33.40	–34.2
MEEA-NP/CTAB	9.1	41.53	35.8
OCT-NP/SDS	7.9	34.33	–34
OCT-NP/CTAB	8.8	40.87	34.9

The oil flooding experiment of the modified alumina nanofluid with surfactants (CTAB and SDS) was conducted in reservoir rocks at 20 °C. The nanofluid tests were based on a previous reported method.^29^ Brine was injected first until the amount of oil displacement was 0.05 mL. This was subsequently followed by testing nanofluids: CTAB, SDS, MEEA-NP/CTAB, MEEA-NP/SDS, OCT-NP/CTAB, and OCT-NP/SDS. The testing parameters, conditions such as pressure, flow rate, temperature, salinity, and pore volume injection, remained the same for all core tests.

**Figure 6:**
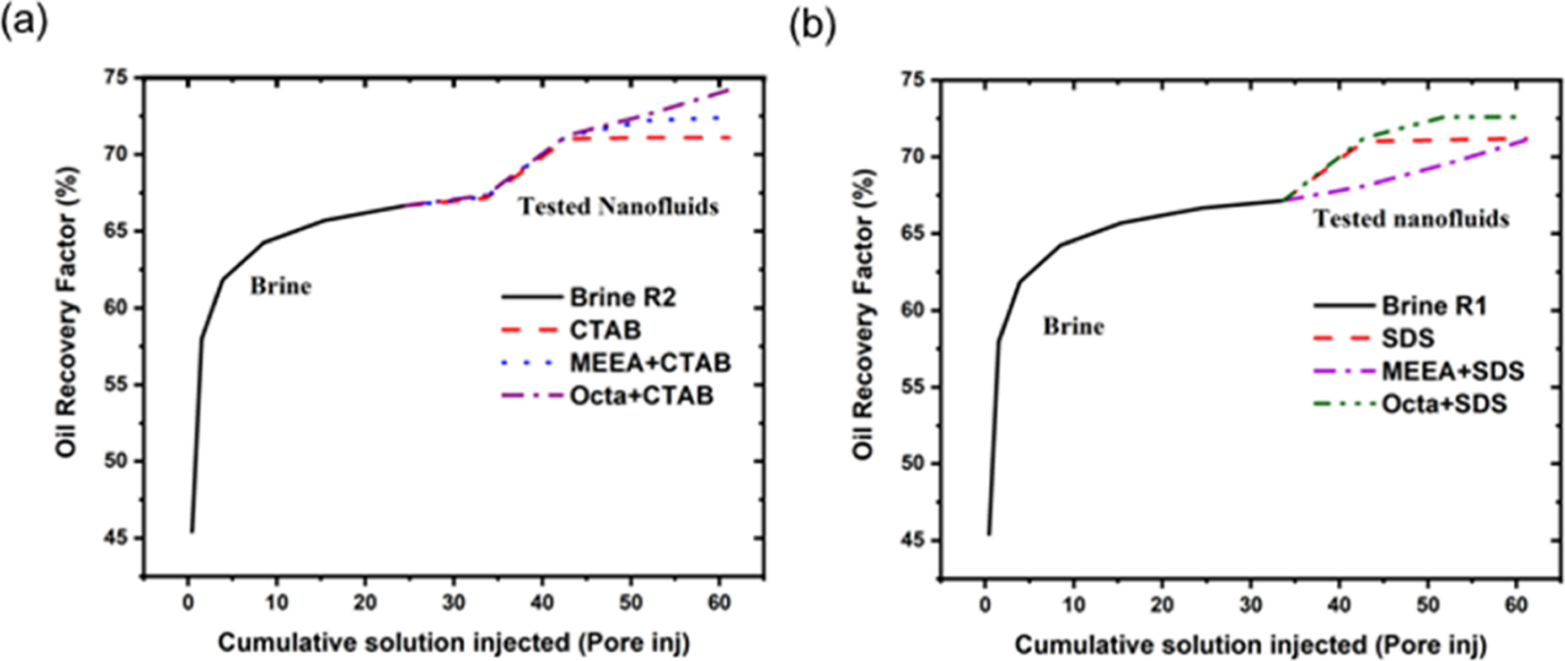
Oil recovery factor in brine solution using sandstone reservoir rocks for MEEA-NPs and OCT-NPs mixing with (a) CTAB surfactant and (b) SDS surfactant.

Figure 6 shows the oil displacement from the injected NPs with the CTAB surfactant (a) and with the SDS surfactant (b). The recovery from NPs dispersed in CTAB solution shows higher percentage than from SDS solution. Interestingly, the recovery from the hydrophobic NPs (OCT-NPs) in both systems showed higher recovery than from hydrophilic NPs (MEEA-NPs). This result is in agreement with our previous research where OCT-NPs showed more effectiveness in oil displacement than MEEA-NPs.^29^ The highest oil recovery was from injected OCT-NP/CTAB, which is around 4% more than the injected surfactant alone as well as the MEEA-NP/surfactant complexes. These results indicate that NP/surfactant complexes can be promising candidates for EOR applications.

## Conclusions

The use of NR has been uniquely applied to obtain information on the behavior of NPs at the oil/water interface. While column tests, ordinarily used by researchers^46^ for the examination of NPs, supply information on the dynamic moving behavior, NR technique provide information on the equilibrium structure and interaction of NPs aggregated and/or deposits at the interface. In this regard, NR is unique compared to other techniques due to its ability to supply information on the distribution of the particles near the interface. In addition, NR provides previously unobtainable information about the interaction between the NPs and surfactants, including the surfactant coverage NPs and their prospective volume fractions. Thus, the introduction of NR as new technique to study particle interaction with surfactant at the interface will advance our current understanding of the mechanisms behind the surface and interaction of NPs with surfactants in natural and engineered environmental systems. The NR data indicated more interaction between NPs and SDS (1:3 ratio); however, the interaction was much smaller (1:15) for NPs with CTAB, with many layers (complex system) at the interface. On the other hand, the adsorbed amount of OCT-NP/CTAB complexes showed more affinity to the oil/water interface compared to the NPs/SDS system, and this has led to a high oil displacement. The IFT data for NPs alone from our previous published paper^29^ showed an IFT of around 40–45 mN/m; however, IFT reduction was significantly improved in the presence of NP/surfactant complexes (8–9 mN/m). This shows that the presence of surfactant and its interaction with NPs significantly improve the surface activity of the NPs, which lead to the reduction of IFT compared to the NPs alone. The oil recovery data showed an increase of around 4% for both OCT-NP (hydrophobic) complexes, especially with CTAB surfactants compared to the surfactant injection alone, which shows that these systems can have a potential use in EOR.

## Supporting Information Available

Additional experimental details of viscosity and density, SLD for testing materials, schematic diagram of contrast A and B, and fitting parameter for INTER and SANS fitting (PDF)

## Abbreviations

NPs: nanoparticles
NR: neutron reflectometry
EOR: enhanced oil recovery
SLD: scattering length density
DLS: dynamic light scattering
QCM: quartz crystal microbalance
AFM: atomic force microscopy
MEEA: 2-[2-(2-methoxyethoxy)ethoxy]acetic acid
OCT: octanoic acid
CTAB: hexadecyltrimethylammonium bromide
SDS: sodium dodecyl sulfate
Si: silicon
SANS: small-angle neutron scattering
CMC: critical micelle concentration
DI: deionized water
IFT: interfacial tension
SFT: interfacial tension
ZPT: zeta potential
