# Supplementary material for: Interaction of Surface-Modified Alumina Nanoparticles and Surfactants at an Oil/Water Interface: A Neutron Reflectometry, Scattering, and Enhanced Oil Recovery Study
Source: ACS Appl Mater Interfaces. 2022 Apr 20;14(17):19505–14. doi: 10.1021/acsami.2c02228 (PMC9096789; doi:10.1021/acsami.2c02228)
Supplement: Supplementary file 1 — am2c02228_si_001.pdf [file am2c02228_si_001.pdf]

Interaction of Surface Modified Alumina Nanoparticles and Surfactants at an Oil/Water Interface: A Neutron Reflectometry, Scattering and Enhanced Oil Recovery Study

Wafaa Al-Shatty,[*†,‡] Mario Campana,[§] Shirin Alexander,[*†] and Andrew R. Barron[*†,ξ,ϕ,φ]*

[†] Energy Safety Research Institute (ESRI), Swansea University, Bay Campus, Swansea SA1 8EN, UK.

[‡] Laboratory and Quality Control Department, Basrah Oil Company, Bab Al Zubair, Basrah 21240, Iraq.

[§] Science and Technology Facilities Council (STFC), ISIS Neutron and Muon Source, Rutherford Appleton Laboratory, Didcot OX11 0QX, UK.

[ξ] Arizona Institute for Resilient Environments and Societies (AIRES), University of Arizona, Tucson, Arizona 85721, USA.

[ϕ] Department of Chemistry and Department of Materials Science and Nanoengineering, Rice University, Houston, Texas 77005, USA.

[φ] Faculty of Engineering, Universiti Teknologi Brunei, Brunei Darussalam.

Table S1. Viscosity and density for fluids formation.

Materials	Viscosity (cp) at ˚C			Density (g/cm^3) at ˚C		
	10	20	30	10	20	30
Oil	5.511	4.511	3.497	0.834	0.827	0.819
Brine	1.751	1.460	1.204	1.141	1.136	1.131
CTAB	1	0.865	0.799	1.001	0.996	0.997
SDS	1	0.875	0.789	1.002	0.997	0.997
MEEA-NP/SDS	1.08	0.97	0.86	1.004	0.999	0.997
MEEA-NP/CTAB	1.1	0.97	0.88	1.005	0.999	0.9965
OCT-NP/SDS	1.23	1.18	0.99	0.988	0.997	0.996
OCT-NP/CTAB	1.25	1.15	0.98	0.987	0.9968	0.9937

Table S2. Values for scattering length density ρ for all components used in the study.

Material	ρ / ×10^{-6} Å^{-2}
Silicon	2.07
H_2O	-0.56
D_2O	6.35
h-SDS	0.34

d-SDS	5.72
h-CTAB	-0.35
d-CTAB	6.24
MEEA-NP and OCT-NP	4.80

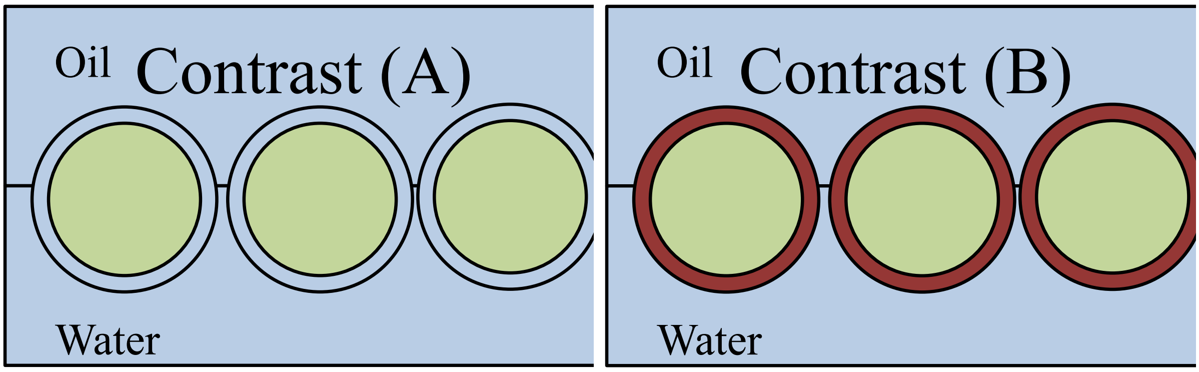

Figure S1. Contrast scheme used in the experiment. In all cases the oil and the water are both matched to silicon. In Contrast (A) a mixture of deuterated- and hydrogenated-surfactant with the same scattering length density of silicon is used. In Contrast (B) the surfactant used is chain deuterated.

Contrast matching calculation

$$SLD\ (h\ and\ d) system = volume\ fraction\ of\ (h) * SLD\ (h) + volume\ fraction\ of\ (d) * SLD\ of\ (d)$$

$$g\ of\ h\ (material) = volume\ fraction\ of\ (h) * density\ of\ (h)$$

$$g\ of\ d\ (material) = volume\ fraction\ of\ (d) * density\ of\ (d)$$

where h refer to hydrogenated material, d to deuterated material, and g to weight in gram.

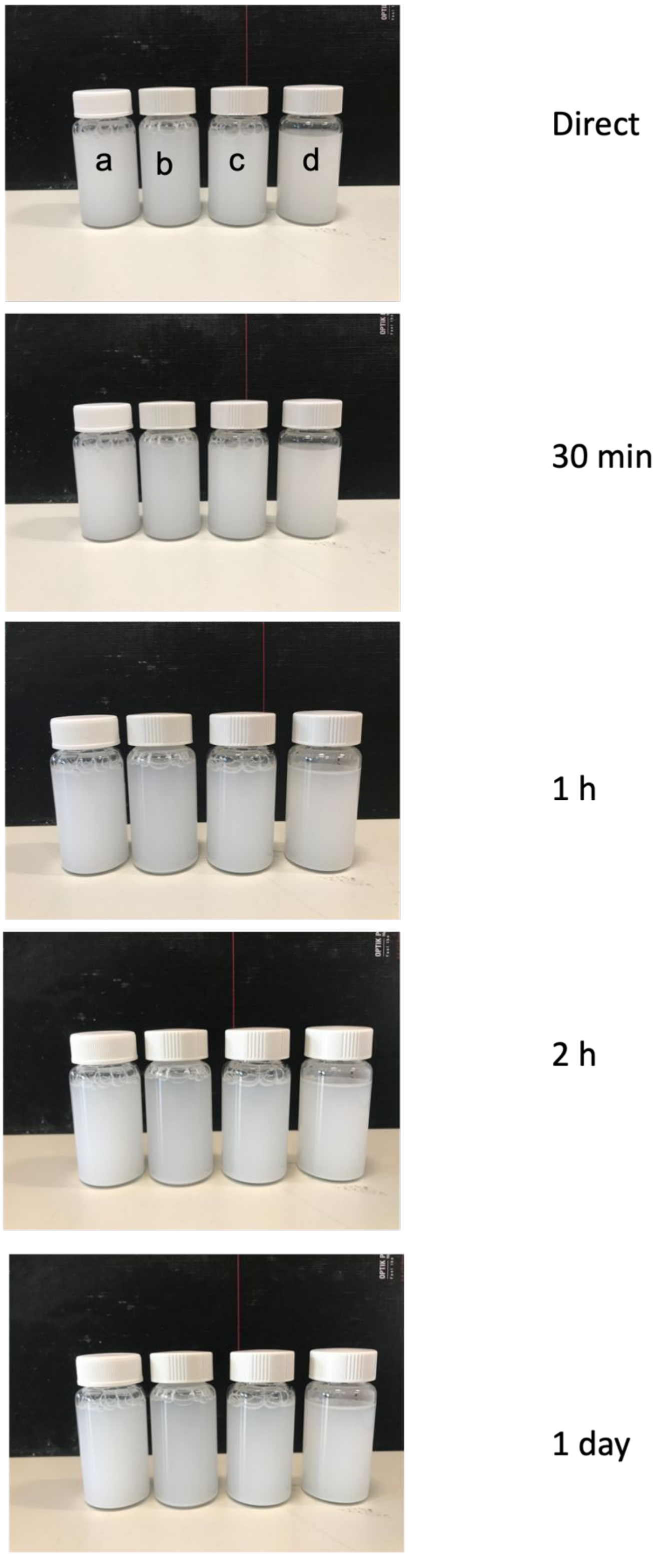

Figure S2. Nanoparticle's surfactant mixtures: a) OCT-NP in SDS surfactant, b) OCT-NP in CTAB surfactant, c) MEEA-NP in SDS surfactant, and d) MEEA-NP in CTAB surfactant.

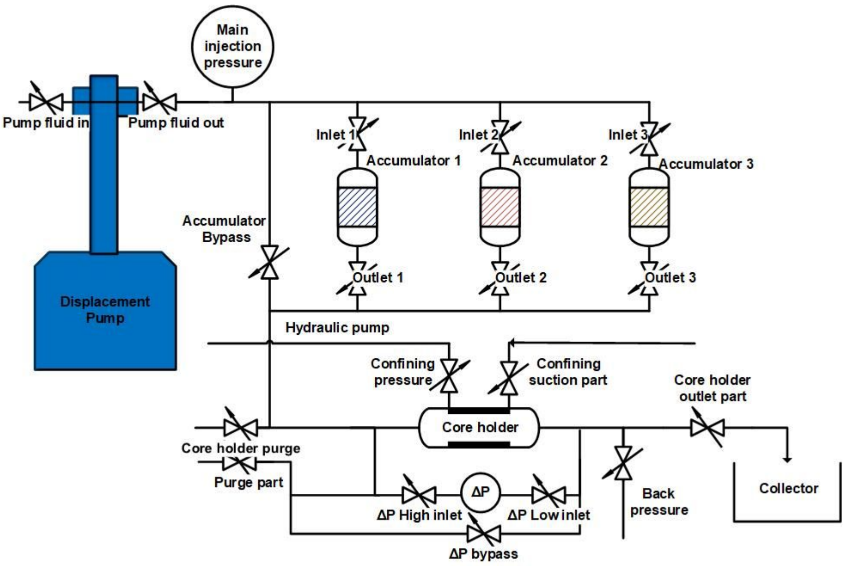

Figure S3. Schematic of oil displacement experiment.

Model description

As clearly stated in the text above, the model used for describing the data differs substantially between NP-SDS and NP-CTAB systems. The two are discussed separately.

NP-CTAB: The interface was initially represented using a 1 layer model characterised by a roughness, bottom roughness, layer thickness (*t*) and hydration (which is dependent of volume fraction of NP, Φ_{NP} and volume fraction of SDS, Φ_{SDS}). The model was deemed too simple and therefore it was decided to adopt the *geometric model*. For this geometric model the NP-SDS complexes were divided into 10 slices composed of 2 concentric ellipsoids: the inner ellipsoid represents a NP with dimensions 42 x 400 x 400 Å. The outer shell has a

thickness of 42 Å. The thickness was the same for each of the slices and was allowed to flow in the fitted procedure. The interlayer roughness was also the same for all layers and fixed at a half the fitted layer thickness. Other fitting parameters were the *surfactant coverage* in the shell (0 = no surfactant, 1 = full coverage) and the *sphere packing* of complexes at the interface (0 = no complexes, 0.909 = full packing). The volume fraction of NPs and SDS in layer *n* was represented as:

$$\Phi_{NP\_Ln} = \text{Sphere\_packing} * Vol_{NP\_Ln}$$

$$\Phi_{SDS\_Ln} = \text{Sphere packing} * \text{Surfactant coverage} * Vol_{SDS\_Ln}$$

Where Vol_{NP_Ln} and Vol_{SDS_Ln} are the volume fractions of NP and SDS in each slice *n* of the complex. These calculated parameters are given in Table S3.

Table S3. Shows the volume fraction of NP and SDS in each slice of the NP-SDS complex.

Slices / *n*	Vol_{NP_Ln}	Vol_{SDS_Ln}
1	0	0.1867
2	0.0386	0.4681
3	0.4063	0.3404

4	0.7226	0.1841
5	0.8807	0.106
6	0.8807	0.106
7	0.7226	0.1841
8	0.4063	0.3404
9	0.0386	0.4681
10	0	0.1867

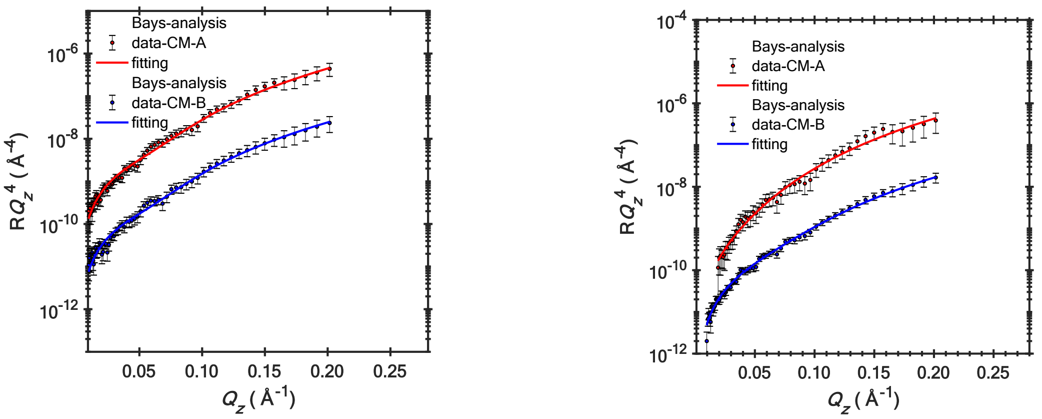

Figure S4. Data and best line fits for (left) the MEEA-NP/SDS and (right) the OCT-NP/SDS system systems using a single layer model. The shaded areas correspond to the 95% confidence interval as determined by Bayesian analysis. Contrast (A) is shown on top, Contrast (B) below. Profiles are offset to visualise the quality of the fit. Because of the low signal measured, data for Contrast (A) contains a few points with zero counts. As Rascal has difficulties simulating data in this situation, the data at Q < 0.02 Å-1 was truncated. The fit describing the whole Q profiles is satisfactory and is shown in Figure S9.

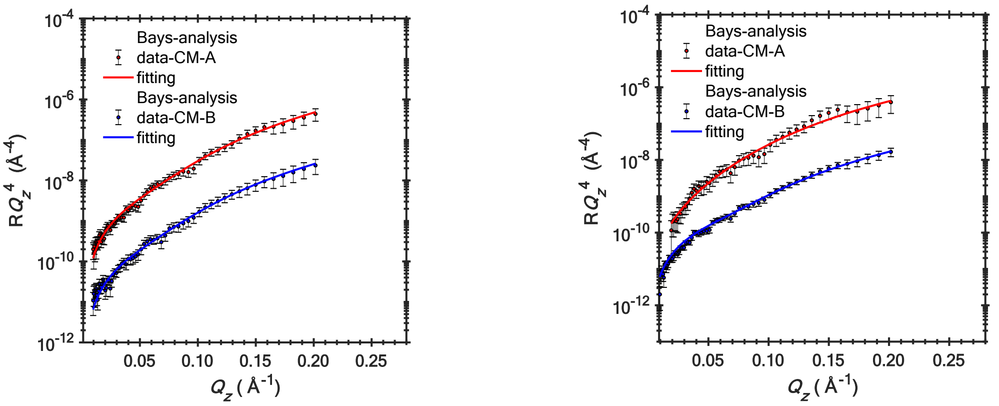

Figure S5. Data and best line fits for the (left) MEEA-NP/SDS and (right) OCT-NP/SDS systems using the near-sphere geometric model. The shaded areas correspond to the 95% confidence interval as determined by Bayesian analysis. Contrast (A) is shown in red and contrast (B) in blue.

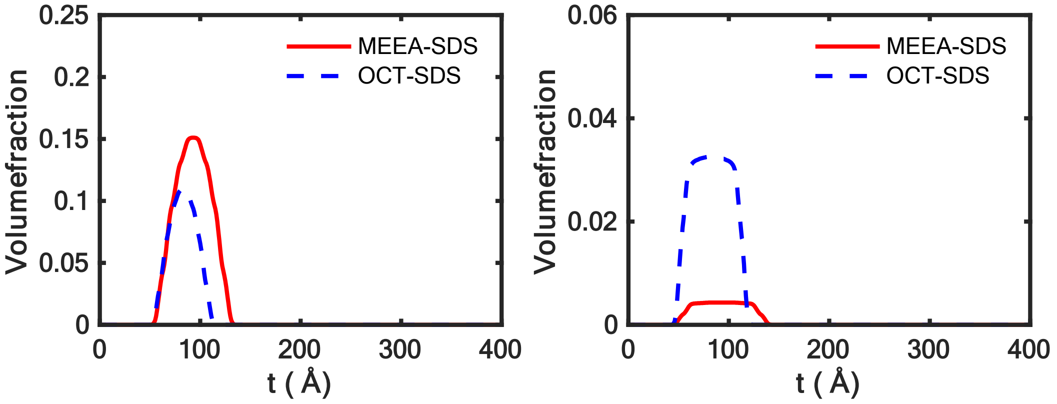

Figure S6. The distribution of NP (left) and surfactant (right) across the interface for both systems for the NP/SDS system using near-spherical particles model. The scaling for the NP and surfactants is different to facilitate visualizing the higher amount of adsorbed NP compared to the surfactant.

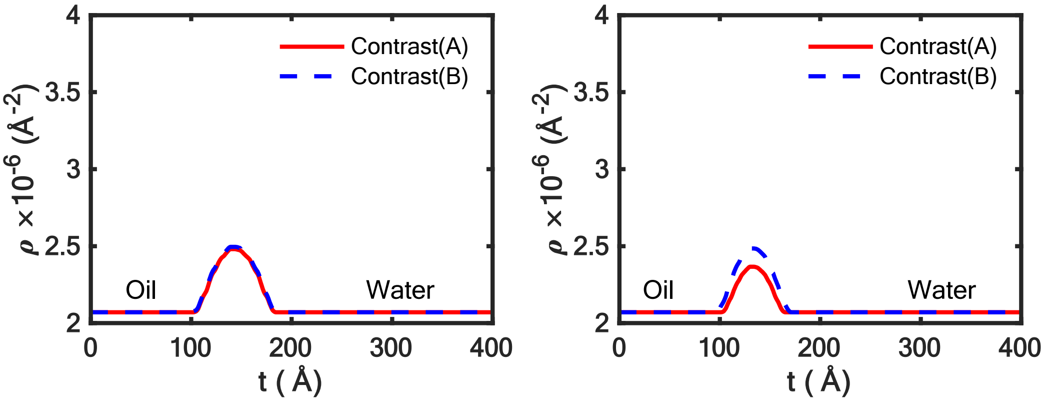

Figure S7. The scattering length density profiles for (left) MEEA-NP/SDS, (right) OCT-NP/SDS using near-spherical particles model. Contrast (A), with CMSi surfactant, is shown in red while Contrast (B), with d-surfactant, is shown in blue. Note that in all cases Contrast (A) has a lower signal compared to Contrast (B).

Table S4. Adsorbed amount for NPs and surfactants in the SDS (near-spherical and Ellipsoidal) systems studied. Surfactant and NPs are shown separately for each system. In all cases the total optimum adsorbed amount is shown together with the 95% confidence interval in parentheses.

Layer	Adsorbed amount, Γ (mg.m^{-2})	
	Nanoparticle	Surfactant
MEEA-NP/SDS (near sphere)	0.732 (0.687,0.792)	0.033 (0.003,0.079)

MEEA-NP/SDS (Ellipsoidal)	0.755 (0.676,0.852)	0.035 (0.0026,0.088)
OCT-NP/SDS (near sphere)	0.404 (0.344,0.477)	0.193 (0.119,0.308)
OCT-NP/SDS (Ellipsoidal)	0.416 (0.360,0.495)	0.154 (0.097,0.247)

NP-CTAB: The interface could be represented using a 3 layer model. Each layer is characterised by a roughness, layer thickness (*t*) and hydration. For each layer the hydration is calculated from the volume fraction of NP Φ_{NP} and volume fraction of CTAB Φ_{CTAB}. For OCT-NP/CTAB the fitted Φ_{CTAB} for the second layer was lower than for the third layer. This was deemed unphysical given the distribution of the OCT- NP, therefore Φ_{CTAB} for layer 2 was constrained to be higher than for layer 3.

$$\Phi_{CTAB\_L2} = Incremental\_\Phi_{CTAB\_L2} + \Phi_{CTAB\_L3}$$

This ensures that the number of fitting parameters does not change between the 2 systems.

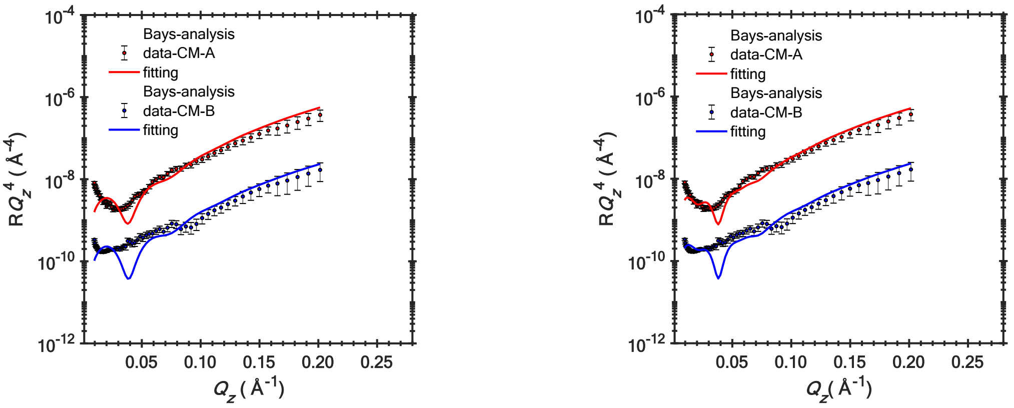

Figure S8. Data and best line fits for the (left) OCT-NP/CTAB system using a 1-layer model in which the fit quality was extremely poor and thus no Bayesian analysis was performed, and (right) data and best line fits for the OCT-NP/CTAB system adopting a 2-layer model. Again, the fit is far from satisfactory and increasing complexity was required to adequately model the interface.

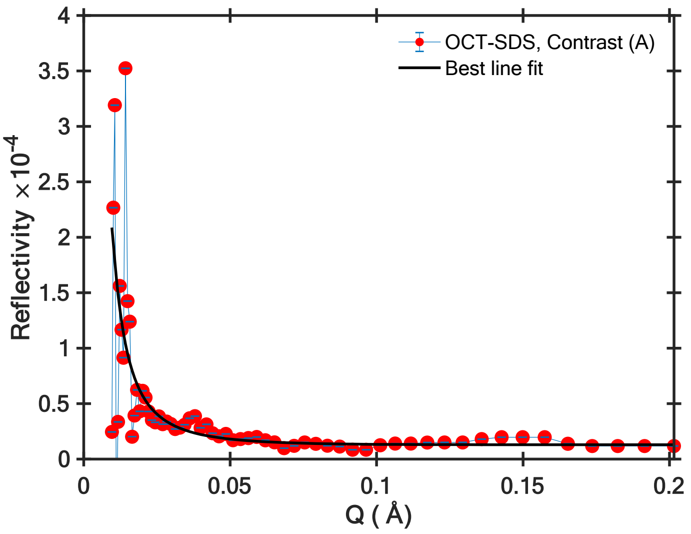

Figure S9. Reflectivity profile and best fit for Contrast (A) for OCT-NP/SDS showing the full Q range.

Table S5. The fitting parameters for MEEA-NP and OCT-NP in SDS surfactant system under study. Each parameter shows the associated best fit parameter next to it, followed by the 95% confidence interval in brackets. Where L refer to layer.

Properties of NR with SDS	MEEA-NP	OCT-NPS
Substrate Roughness	8.08 (1.31, 12.84)	7.37 (1.4383, 12.72)
Thickness_L1	48.37 (37.14, 59.69)	38.63(27.5, 47.93)
Vol-NP_L1	0.15(0.15, 0.19)	0.11 (0.085, 0.157)
Vol-SDS_L1	0.007 (0.0008, 0.013)	0.044(0.032, 0.06)
Roughness_L1	7.63 (0.17, 12.86)	6.93 (0.51, 12.68)

Table S6. The fitting parameters for MEEA-NP and OCT-NP in CTAB surfactant system under study. Each parameter shows the associated best fit parameter next to it, followed by the 95% confidence interval in brackets. Where L refer to layer.

Properties of NR with CTAB	MEEA-NP	OCT-NPS
Substrate Roughness	6.23 (2.35, 23.27)	7.147 (1.46, 12.62)
Thickness_L1	33.01(25.51, 4.40)	33.67 (25.49, 43.91)
Thickness_L2	158.94 (147.38, 169, 05)	174.92 (157.54, 189.29)
Thickness_L3	177.69 (161.16, 193.38)	180.74 (167.18, 193.77)
Vol-NP_L1	0.396 (0.34, 0.45)	0.35 (0.302, 0.42)
Vol-NP_L2	0.25 (0.24, 0.26)	0.24 (0.22, 0.25)
Vol-NP_L3	0.0312 (0.019, 0.26)	0.047 (0.034, 0.061)
Vol-CTAB_L1	0.0364(0.019, 0.052)	0.0979 (0.067, 0.13)
Vol-CTAB_L2	0.0108 (0.003, 0.018)	0.0006 (0.00, 0.003)
Vol-CTAB_L3	0.005 (0.0001, 0.015)	0.009 (0.008, 0.012)
Roughness_L1	6.735 (0.520, 12.7)	7.22(0.53061, 12.78)
Roughness_L2	72.195 (65.795, 74.893)	72.19 (65.60, 74.91)
Roughness_L3	18.65 (2.45, 28.24)	21.46 (5.60, 31.39)

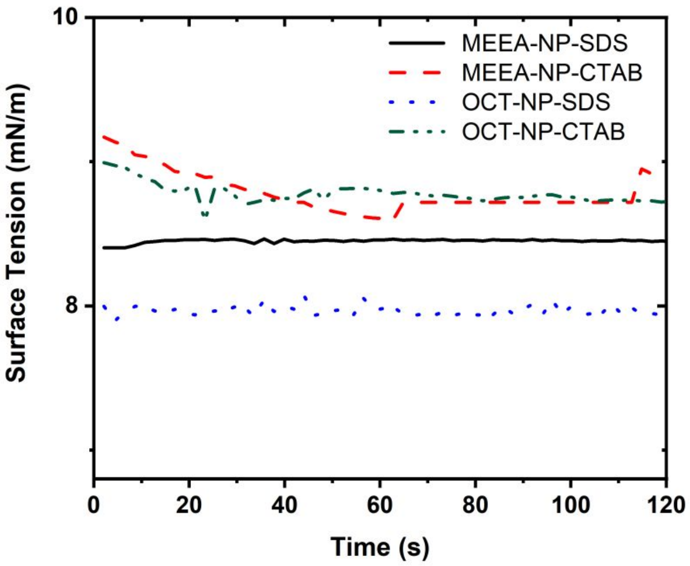

Figure S10. Dynamic IFT of MEEA-NP and OCT-NP in SDS and CTAB surfactant in n-hexadecane at 120 s.
